# Sediment biomarker, bacterial community characterization of high arsenic aquifers in Jianghan Plain, China

**DOI:** 10.1038/srep42037

**Published:** 2017-02-06

**Authors:** Hengpeng Ye, Zeyu Yang, Xiang Wu, Jingwen Wang, Dongyun Du, Jian Cai, Kangle Lv, Huiyun Chen, Jingkun Mei, Mengqi Chen, Hong Du

**Affiliations:** 1 School of Resource and Environment Science, Key Laboratory for Catalysis and Materials Science of the State Ethnic Affairs Commissions & Ministry of Education, South Central University for Nationalities, Wuhan 430074, China; 2 Emergencies Science and Technology Section, Science and Technology Branch, Environment and Climate Change Canada, Ottawa, ON, Canada

## Abstract

Representative biomarkers (e.g., *n*-alkanes), diversity and microbial community in the aquifers contaminated by high concentration of arsenic (As) in different sediment depth (0–30 m) in Jianghan Plain, Hubei, China, were analyzed to investigate the potential mechanism of As enrichment in groundwater. The concentration of As was abundant in top soil and sand, but not in clay. The analysis of the distribution of *n*-alkanes, CPI values, and wax to total *n*-alkane ratio (Wax(n)%) indicated that the organic matter (OM) from fresh terrestrial plants were abundant in the shallow sediment. However, *n*-alkanes have suffered from significant biodegradation from the depth of 16 m to 30 m. The deposition of fresh terrestrial derived organic matters may facilitate the release of As from sediment to groundwater in the sediment of 0–16 m. However, the petroleum derived organic matters may do the favor to the release of As in the deeper section of borehole (16 m to 30 m). The 16S rRNA gene sequences identification indicated that *Acidobacteria, Actinomycetes* and *Hydrogenophaga* are abundant in the sediments with high arsenic. Therefore, microbes and organic matters from different sources may play important roles in arsenic mobilization in the aquifers of the study area.

Arsenic (As) contamination of groundwater is a problem that affects millions of people across the world^1^, which is related to arsenic poisoning, such as heart disease, cancer, stroke, chronic lower respiratory diseases, and diabetes^2^,^3^.

Previous studies on the aquifers contaminated by high level of arsenic have indicated that the major source of As in groundwater is released from the sediment^4^,^5^,^6^,^7^,^8^. As resistant microbes interacting with various geochemical processes, play an important role for the mobilization and transformation of As in aquifers and groundwater^9^,^10^. Arsenic may be mobilized from arsenic bearing iron oxides/hydroxides within aquifer sediments into groundwater, as organic matter (OM), especially for labile redox-active components of the OM, e.g., humic substances, plays a critical role in increasing rates of microbial-mediated iron reduction and ground arsenic mobilization^11^,^12^ Therefore, study the characteristics of organic matters (OM) is very important for understanding the mechanism of arsenic mobilization in arsenic-contaminated aquifers. Biomarker analysis can provide useful clues about microbial activity in aquifer sediments^13^,^14^.

The mechanism for arsenic-transforming microbes facilitating the release and mobilization of arsenic from aquifers to groundwater have been reported previously^15^,^16^. For example, *Pseudomonas* species and *Clostridium* species, as the effective metal reducing microbes, were identified from the sediments in Bengal delta, where high arsenic was detected^17^. The relative abundances of different bacteria (such as, *Deltaproteobacteria*, namely *Geobacter* species, and *Taxa*), have been reported to correlate with iron reduction and arsenic mobilization^15^,^18^,^19^. Except for organic matter quality and quantity, and the concentrations of specific metals affecting the bacterial community structure in high arsenic aquifers^19^, bacterial community structure may be specific in different environmental conditions^20^.

In 2005, the first case of arsenic poisoning in the Jianghan Plain was reported in Shahu Village, Xiantao, Hubei, China. The following survey was followed in May 2006, to assess the distribution of arsenic in groundwater in 19 towns in Xiantao by the Center for Endemic Disease Control of Xiantao and Hubei Province. The results showed that 863 wells in 12 towns (179 villages) had arsenic levels exceeding the China’s National Drinking Water Standard of 10 μg/L^21^. Moreover, arsenic concentrations in some wells were 50 times higher than the standard. Since 2006, much attention has been paid to endemic As poisoning in drinking water in Jianghan Plain, Hubei, China^22^,^23^,^24^,^25^. In this study area, the arsenic concentrations accompanying with the relative ratio of As (III) increase in rainy season, but decrease till the end of dry season due to the inductive mobilization of arsenic^11^,^26^. High arsenic concentrations in groundwater mainly occur between the vertical depths of 10 and 45 m below ground surface. High concentrations of dissolved organic carbon (DOC) in groundwater indicate reducing conditions with abundant organic matter in the groundwater aquifers. Fe and Mn oxides/hydroxides are considered to be the dominant minerals containing As in sediments and the main sources for As in groundwater of the Jianghan Plain. The main potential mechanism for the release of As is the reductive dissolution of Fe and Mn oxides/hydroxides under reducing conditions. This process changes the speciation of Fe from solid phase Fe(III) to soluble Fe(II) with the characteristic of reductant, which may reduce As (V) to the more easily desorbed As (III). Simultaneously, the microbial degradation of organic matter may also facilitate the release of arsenic into groundwater due to the speciation of As reducing to As (III)^11^,^27^. The widespread occurrence of dissolved organic carbon in sediment was reported to contribute to groundwater DOM, promote the reducing conditions of the groundwater and significantly influence the groundwater chemistry^25^.

Until now, most of the reports focused on the study the hydrochemistry of groundwater^11^,^23^,^26^,^27^ to infer the potential mechanism of the occurrence of As in groundwater in Jianghan Plain. The analysis of the diversity and structure of the microbial communities and chemistry in related sediment will help us to further elucidate the potential release mechanism of As.

In this study, three sediment bore cores with length of 20–30 m were drilled in 2013. The vertical distribution profiles of arsenic, representative *n*-alkanes, and bacterial community characterization of several representative high arsenic aquifers in Jianghan Plain of Hubei, China, were investigated to elucidate of the potential role of organic matter and microbial activity on arsenic enrichment in groundwater in the study area.

## Methods

### Regional hydrogeology

Jianghan Plain is the first large-scale river basin receiving sediment from Yangtze River downstream of the three Gorges. Situated in the central and southern regions of Hubei Province, it features Quaternary lacustrine sediments in the middle reaches of the Yangtze River. It has a warm and humid subtropical monsoonal climate, annual mean temperature 15–17 °C, altitude about 50 m, annual rainfall about 1160 mm. The typical characteristics of the geology, hydrogeology, and land use have been discussed in some previous reports^25^,^28^. In brief, the typical topographical structure in this area is a semi-closed basin with a higher elevation in the north, a lower elevation in the south, and a low alluvial plain in middle region. The hilly areas primarily consist of aquitard, while the center consists of unconsolidated water-bearing sediment layers. Clayey silt, sandy silt, sandy clay and interlaced clay lenses, the main lithology characteristic for the unconsolidated sediments in the depth of 10–35 m, compose of the unconfined aquifer in the study area. The depth of groundwater level is about 0.5–2.0 m. The unconfined aquifer is recharged by precipitations during the rainy period, as well as by surface waters when situated close to them. Evaporation, drainage of the rivers and leakage to underlying formations are mainly responsible for the discharge of the unconfined aquifer. Pliotstocene sand and sandy gravel consist of the confined aquifer. Recharge of the confined aquifer occurs during floods season by infiltration of rivers and leakage from the phreatic water zone where the impermeable clay layer is thin enough. Discharge of the groundwater occurs through regional flow. There is no such a heavy exploitation of the aquifers for human activities (industries and agriculture) that may affect the natural regime of the groundwater (Hubei Hydrogeology and Geology engineering station, 1985 cited in ref. 29).

### Sampling site description and sample collection

**Figure 1:**
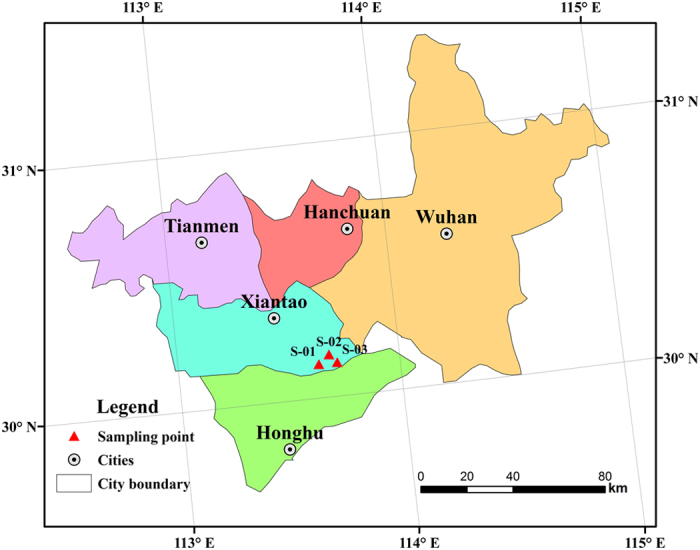
Map illustrating the sampling sites in the Jianghan Plain, China (Created by using Adobe Photoshop CC (serial number: 9706-8123-3218-6079-7613)).

Three sediment bore cores with a total depth of 20–30 m were drilled by rotary drilling in March 2013 (S–01, 30°08′N/113°38′E; S–02, 30°10′N/113°41′E; S–03, 30°08′N/113°43′E, Fig. 1). The land in the sampling area is mainly used for paddy and wheat fields. The three sampling sites locate in the interior of the low alluvial plain; the sites are surrounded by rivers, and covered by other abundant surface water bodies such as ponds, irrigation channel, and wetlands. Strong surface water-groundwater interactions are observed here^11^. Samples from the core of S–01 was designated as the control samples as the As concentration in the groundwater in this sampling site is 5 μg/L, which meets the standard criteria specified by China. The groundwater As concentrations in S–02 and S–03 were determined to be 1560 and 1340 μg/L, respectively. The representative chemical composition of groundwater is this study area will be discussed in the following section. Samples were sectioned at every 2 to 4 m on site. Sectioned cores were wrapped with the pre-cleaned and verified aluminum foil, capped immediately with PVC pipe, and kept in *N*_2_ atmosphere in −20 °C, to minimize the exposure of sediments to atmospheric oxygen and microbial degradation of organic matters.

### Arsenic analysis

Sediment samples were first homogenized with a mortar and pestle, and passed through a 200 μm sieve to remove plant roots and miscellaneous debris. A small amount of sample, 0.1 g, diluted with 1 mol/L phosphoric acid and 0.5 mol/L ascorbic acid, was microwave digested by a method adopted from ref. 30 to extract arsenite and arsenate. The total arsenic in the digested extracts was analyzed by hydride generation atomic fluorescence spectrometry (HG-AFS) (AFS-820, Titan). The relative standard derivation for arsenic measurement was less than 10% in the present study.

### Representative biomarker analysis

An aliquot (around 10 g dry weight, d. w.) of homogenized sample mixed with sodium sulfate, spiked with [^2^H]_50_ tetracosane (*C*_24_*D*_50_) and deuterated polycyclic aromatic hydrocarbon (PAH) mixture as surrogates, including [^*2*^*H*_8_] naphthalene (naphthalene-d_8_), [^*2*^*H*_10_] acenaphthene (acenaphthene-d_10_), [^*2*^*H*_10_] phenanthrene (phenanthrene-d_10_), [^*2*^*H*_12_] benz(a)anthracene (benz(a)anthracene-d_12_), and [^*2*^*H*_12_] perylene (perylene-d_12_), was Soxhlet extracted with dichloromethane (DCM) for 24 h. Extracts were concentrated and solvent-exchanged into hexane. Approximately 2 g of activated copper was added for desulphurization overnight. The extracts were then quantitatively transferred into a 3-g of silica gel chromatography column topped with about 1-cm anhydrous granular sodium sulfate for cleanup and fractionation. Hexane (12 mL) and 50% DCM in hexane (v/v, 15 mL) were used to elute the saturated and aromatic hydrocarbons, respectively. The hexane fraction was used for analysis of *n*–alkane. These two fractions were concentrated under a gentle stream of nitrogen to appropriate volumes, spiked with appropriate internal standard (IS), e.g., 5-α-androstane, for *n*-alkane analysis, and then adjusted to an accurate pre-injection volume of 1.00 mL for gas chromatography/mass spectrometry (GC/MS) analyses.

The hydrocarbons were then analyzed by GC–MS (Agilent 6890 N/5975 MS), equipped with an autosampler. A HP-5 MS capillary column (30 m × 0.25 mm × 0.25 μm) was used. The operating conditions were as follows: oven temperature increased from 80 to 290 °C at 5 °C/min, finally held at 290 °C for 30 min. Samples were injected in splitless mode (injector temperature at 290 °C) with helium as carrier gas. The MSD was operated in the selected ion monitoring (SIM) mode. Agilent Enhanced MSD ChemStation software was used for system control and data acquisition. The identification of compounds was based on the authentic standards, published literature and the NIST chemical data library. The quantification was made by comparing individual peak area with that of a known concentration of internal standard of 5α-androstane. Sodium sulfate as blank control samples were also analyzed following the same procedures as the sediment samples.

### Diagnostic ratios of *n*-alkanes

Diagnostic ratios (e.g., carbon preference index (CPI)) have been developed and applied to identify the origin of *n*-alkanes^31^. CPI_1_ is usually used to discriminate *n*-alkanes between petrogenic and biogenic sources, which is defined by the ratio between the sum of all *n*-alkanes from *n*-*C*_9_ to n-*C*_40_ with odd carbon number and the sum of those with even carbon number (equation 1). Generally, CPI_1_ around 1 suggests petrogenic input; naturally generated hydrocarbons link to higher plants exhibit values of CPI_1_ > 1, usually 5–10^31^. CPI_2_ is utilized to distinguish the fraction of *n*-alkanes from high plant wax, which is calculated by the ratio between all the *n*-alkanes with odd carbon number from *n-C*_27_ to *n*-*C*_36_ and the sum of those with even carbon number (equation 2). High CPI_2_ value is an indicative of high plant wax input^32^. Similarly, plant wax alkanes (WaxCn (%), equation 3) have been used to estimate the contributions from allochthonous and autochthonous organic matter inputs. WaxCn(%) is close to zero for petroleum or crude oil residue, whereas, WaxCn(%) approaches to 100 for high terrestrial plants or marine plants^33^,^34^.


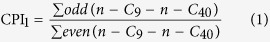



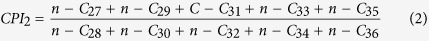



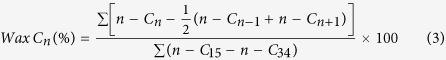


### DNA extraction and clone library construction

Four representative sediment samples (0.25 g) from S–02 and S–03 were extracted using a Fast DNA spin kit (PowerSoil^®^DNA Isolation Kit, MO BIO Laboratories) to get DNA extracts.

PCR: The 16S rRNA gene was amplified from the DNA extracts using universal primers 27 f (5′-AGRGTTTGATCMTGGCTCAG-3′) and 1492 R (5′-GGTTACCTTGTTACGACTT-3′). The purity of the amplified product was determined by the electrophoresis of 10 μL of samples in a 1.0% agarose tris-borate-EDTA (TBE) gel. DNA was stained with ethidium bromide and viewed under short-wave ultra-violet (UV) light.

Amplicon pools from four environments were subjected to cloning as follows: Amplicons were cleaned up and chemically bond into pGEM-T Easy vector using the manufacturer’s protocol (Promega, Fitchburg, WI). The recombinant plasmids were used to transform competent *Escherichia coli* JM109 cells, which were plated on Luria-Bertani plates containing 100 μg/mL of Ampicillin, 80 μg/mL of 5-bromo-4-chloro-3-indolyl-*β*-D-galactopyranoside (X-Gal), and 0.5 mmol/L of isopropyl-*β*-D-thiogalactopyranoside (IPTG) and incubated overnight at 37 °C. Randomly selected white clones were screened by colony PCR amplification of the 16S rRNA gene inserts using M13 primers.

### Nucleotide sequence accession numbers

Positive clones were randomly picked and sequenced by TsingKe Biological Technology. The raw sequences were trimmed by using DNAman 6.0. The resulting sequences were classified by RDP online (http://rdp.cme.msu.edu/). Phylogenetic and statistical analyses were performed with Bioedit 7.0.9, MEGA 5.02. Sequences above 97% identity were defined as one operational taxonomic unit (OTU), with the resulting 16S rDNA sequences (about 850 bp) in each OTU compared with those in GenBank database using BLAST. The most similar 16S rRNA sequences in each OTU from the GenBank databases were chosen to construct phylogenetic trees. Rarefaction analysis was used to evaluate the saturation of the sampled clones by EstimateS 9.1.0.

Coverage (C) value was calculated to evaluate the representative of the analyzed clones for species diversity in samples according to the formula: C = (1 − n/N) × 100%, where n is the number of 16S rDNA types appearing only once in the library; N is the total number of positive clones detected.

## Results and Discussion

### Physicochemical characteristic of the groundwater in the study area

The physicochemical parameters, including As, Cl^−^, SO_4_^2−^, NO_3_^−^, HCO_3_^−^, Mn, Fe and dissolved organic carbon (DOC), pH and Eh, for groundwater samples in the average depth of 10–45 m were analyzed and reported in ref. 27. In brief, the groundwater in the study area is mainly HCO_3_-CaMg type with circum-neutral pH and moderate to high electrical conductivity. Negative Eh and high concentration of DOC indicate the reducing conditions with abundant organic matter in the groundwater aquifers. High As concentrations (up to 2330 μg/L) were detected in groundwater sampled in the study area, where 87% of them exceed the WHO recommended value of 10 μg/L. High concentrations of dissolved Fe, Mn, and P were also observed in groundwater, with 89% and 98% of them exceeding the WHO guideline for Fe and Mn. Similarly, 60 groundwater samples in the wells within the depth range of 20–30 m were collected and analyzed for their physicochemical characters in 2011 by our group. The concentrations of As range from non-detectable to 1560 μg/L, with 63% of the samples exceeding the WHO guideline limit and 25% of them >50 μg/L. The pH values range from 6.1 to 7.2, with an average value of 6.6. The DOC contents were determined to range from 2.4 to 5.3 mg/L, with an average values of 3.3 mg/L. Positive correlation was observed between the levels of As and Cl^−^, HCO_3_^−^, Mn, Fe, while the levels of SO_4_^2−^, and NO_3_^−^ are low in the high As groundwater.

### Lithology of sediment cores

**Figure 2:**
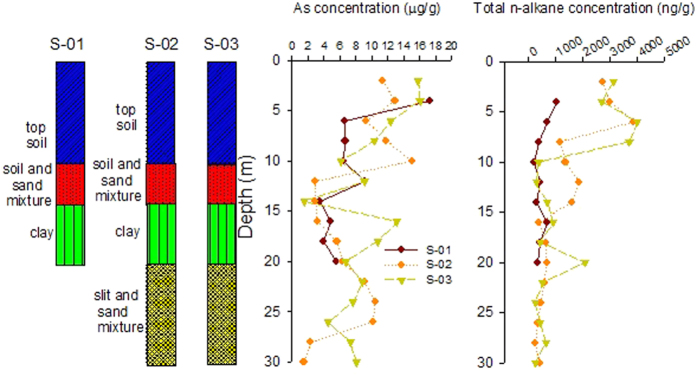
Vertical variation of lithology, total arsenic and total *n*-alkanes with sediment depth in the three studied bore cores.

The sedimentary sequences altering from clays to sand for the boreholes of S–02 and S–03 follow very similar patterns (Fig. 2). The top layer in the two boreholes consists of a soil cap (0–0 m), where the soil is brown in color in the surface (0–2 m) with abundant organic matters, then change to red brown and light brown till to 10 m depth. This phenomenon indicates the limit of O_2_ ingress from the surface^14^. The remaining soils mix with sands to make the depth of 10 to 14 m with a grey to brown color. Then clay is dominant from the depth of 14 to 20 m with a grey color. Below this layer, the sediments predominantly consist of the mixed silt and sands with color ranging from dark grey to dark from 20 to 30 m. These silt and sediments are suggestive of deposition in a fluvial environment, which is consistent with the environment in this study area. Sediment samples from the control borehole S–01 also consist of top soil, mixed soil and sand, and clay, but the color varies from yellow to grey from the surface to bottom, which may primarily indicates less organic matters present in the top soil layer compared to the brown color of the top soil in S–02 and S–03. It is noted that the depth of the borehole of S–01 is only 20 m deep, while the others are 30 m. In conclusion, all the three boreholes show similar sediment lithology, despite that S–01 only has 20 m long, and the color of its top soil is lighter than the other two.

### Vertical variation of total arsenic with sediment depth and lithology

The total arsenic concentrations in the S–01, S–02 and S–03 range from 3.5 to 17.3 μg/g, 1.5 to 15.0 μg/g, and 1.5 to 16.0 μg/g, respectively. Some similarities were observed by analyzing the vertical distribution of As among the three boreholes, although differences are also present. The As contaminations vary at different deposition depth in the three boreholes (Fig. 2). Specifically, the As concentrations in the borehole of S–01 range from 3.5 to 9.1 μg/g from the depth of 20 m to 6 m with a slightly increasing trend at the depth of 12 m. It can be seen that all these values are lower than the WHO limit of 10 μg/L. It increases to 17.3 μg/g in the top soil layer (from 4 m to the surface). In the borehole of S–02, As is low in the depth of 30 to 28 m (the mixed silt and sand layer), while it increases to 10 μg/g in the mixture of silt and sand layer (from 26 to 24 m); then it decreases to around 3 μg/g in the clay and the mixed soil and sand layer (20 m to 12 m), followed by an increasing trend in the top soil layer (10 m to the surface). In the S–03 bore hole, it fluctuates from 4.6 to 13.2 μg/g for the depth of 30 to 20 m, which is characterized as the mixed silt and sands; lowest As (1.5 μg/g) can be observed in the depth of 14 m (clay layer); then an increasing trend (from 6.1 to 16.0 μg/g) is present in the soil and sand mixture layer and top soil layer (from 12 m to surface). It is clear that arsenic level is closely associated with the lithologic structure of the sediment core. Generally, arsenic level is high in the mixed silt and sands and top soil layers, but low in the clay layer locating in the middle of the bore cores. The comparison of the three boreholes indicates that borehole S–01 has the lowest arsenic contamination, while S–02 and S–03 have similar impacts by As. This finding is consistent with the detected As concentrations in the specific groundwater samples. As we have mentioned in the previous sections, groundwater samples from the well close to S–01 has the lowest As, while those from the sites of S–02 and S–03 have similar and relatively high As contamination. This consistency also primarily indicates that As in sediments are the main sources for As in groundwater of the Jianghan Plain^27^.

### Vertical variation of total *n*-alkanes in the sediment cores

Figure 2 also depicts the variation of total *n*-alkanes with sediment depth. The contents of total *n*-alkanes range from 209 to 1020 ng/g, 234 to 3872 ng/g and 236 to 4012 ng/g, respectively, in the sediment bore cores of S–01, S–02 and S–03. The total *n*-alkanes in S–01 are generally lower than S–02 and S–03 at the same depth, despite the data from 22 to 30 m are not available. The highest concentrations of *n*-alkanes were detected in the surface for all the three sites. The vertical trends of total *n*-alkanes in S–02 and S–03 are similar to each other, but vary with sediment depth. In detail, *n*-alkane contents almost keep constant from the depth of 30 to 10 m in spite of some variation from the depth of 26 to 20 m, but they increase significantly in the surface soil (0–10 m). Comparing S–01 with S–02 and S–03, similar vertical distribution profile of *n*-alkanes were identified in S–01 from the depth of 20 m to 10 m; S–02 and S–03 have an increased shift from 8–10 m to 6–8 m, while S–01 does not change significantly at the same depth range. The significant increase is present in S–01 for sediments from 4 m to the surface. It seems that the distribution of *n*-alkanes in different depths shows positive relationship to arsenic and soil characteristic. For example, both *n*-alkanes and arsenic are abundant in the top soil layer. Some differences are also present between *n*-alkanes and arsenic. For example, *n*-alkanes under the top soil layer are generally lower than the top soil layer, while significant amount of arsenic was detected in the mixed silt and sand layer. For *n*-alkanes, it is reasonable because top soil in the study area is abundant with OM, which are the left over in the paddy and wheat field each year. The degradation of the plant remains increases the organic carbon with the typical character of terrestrial input in top soil (see the source identification *n*-alkanes in the following section). Accordingly, abundant dissolved organic carbon (DOC) in shallow groundwater has been reported, which may also facilitate the release of As from sediments into shallow wells^11^. Higher *n*-alkanes in top soil, but lower in the deep sediment primary indicates that OM have been suffered from significant microbial degradation or originated from different sources.

**Figure 3:**
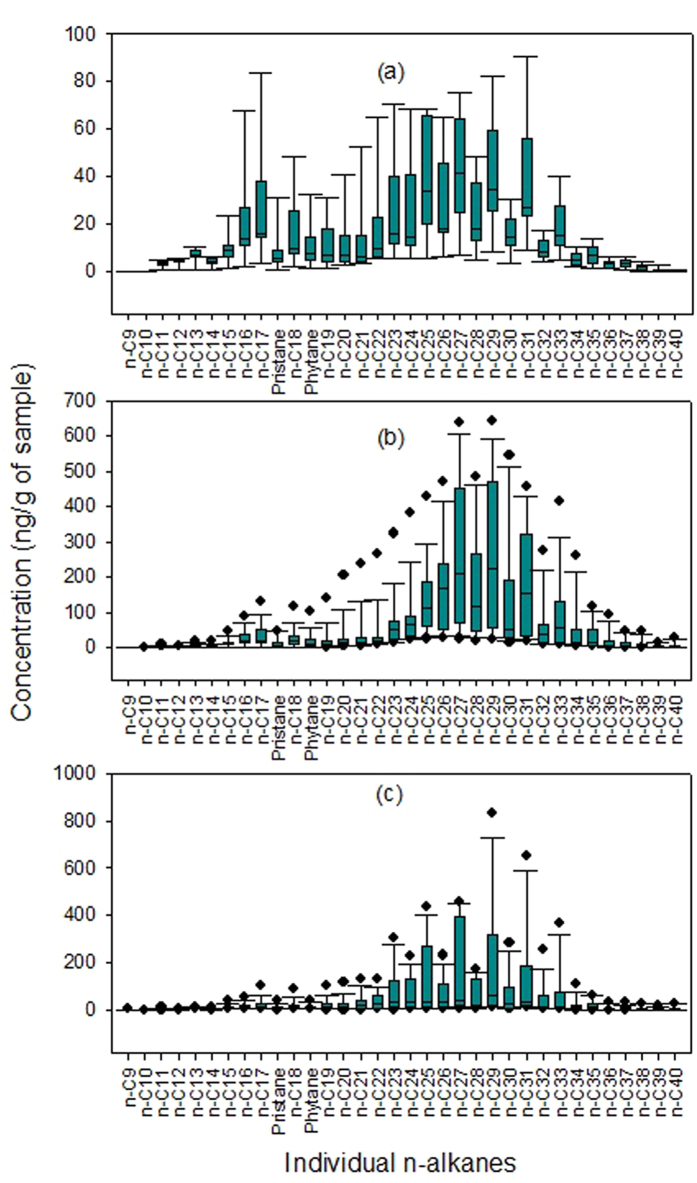
Box plots of *n*-alkanes in the three studied bore cores. (**a**) S–01; (**b**) S–02; (**b**) S–03.

The box plots of individual *n*-alkanes in Fig. 3 depict their full range of variation in the different depths of the three bore cores. Analysis of *n*-alkanes reveals the typical double peak shapes with a general dominance of high molecular weight congeners (*C*_25_–*C*_33_), and obvious odd to even preference in the high number of carbon range. The three bore cores have similar distribution profiles, while their absolute concentrations show some differences. Specifically, S–01 has the lowest *n*-alkanes, while S–02 and S–03 have similar level of them. It is clear that the three bore cores have abundant organic matter input from terrestrial plant, especially for S–02 and S–03. The detailed source identification of *n*-alkanes in the following section will further identify their major source.

### Source identification of *n*-alkanes

**Figure 4:**
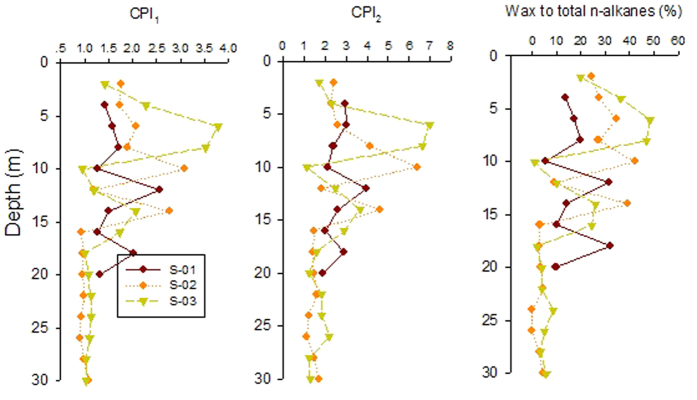
Source identification of *n*-alkanes in the studied sediment bore holes.

Figure 4 depicts the variation of the CPI values and Wax Cn (%) with sediment depth in the three sediment cores. It can be seen that both CPI values are close to 1.0 from the depth of 30 m to 16 m in S–02 and S–03, suggesting the major petrogenic source. However, the two CPI values show an increasing trend from the depth of 16 m to the surface, where CPI_1_ and CPI_2_ are generally higher than 1.0. For example, the maximum CPI_1_ and CPI_2_ are close to 3.5 and 7.0 in the sediments from 16 m to the surface. In S–01, the both CPI values do not have an obvious increasing or decreasing trend with the sediment depth, while CPI_1_ and CPI_2_ fluctuate in the range of 1.3–2.6 and 1.9–4.0 in the whole sediment core. Generally, high CPI_1_ values suggest the predominant biogenic input of *n*-alkanes; high CPI_2_ values indicate the predominant terrestrial plant input^35^. Just as we have mentioned in the above sections, paddy and wheat fields are the typical cultivation character in the study area. The CPI values in the boreholes of S–02 and S–03 shift from around 3.5/7.0 in the sediments form 16 m to the surface to around 1 in the sediments from 30 m to 18 m, indicating a shift from predominantly immature terrestrial derived organic matter to a more diagenetically or thermally mature character, at least of the hydrocarbon component of the OM^14^. While S–01 only has 20 m deep, the >1 CPI values in the whole core also suggest the predominant terrestrial derived organic matter in the study depth. Similarly, the WaxCn (%) contents shift from 42.2% and 48.2% in the sediments (14 m to the surface) to 0 and 1.8% in the sediments deeper than 14 m in S–02 and S–03. Similar to the variation of CPI values, WaxCn (%) contents in S–01 do not have obvious increasing or decreasing trend. The WaxCn (%) in S–01 vary from 5.4 to 32.2% in the 20 m long core, suggesting the predominant immature terrestrial derived OM. It can be concluded that same source of organic matters have contributed to the three boreholes, despite that S–01 do not have obvious input of petroleum derived OM due to the sediments deeper than 20 m were not investigated.

Based on the compound distributions of *n*-alkanes and the diagnostic ratios analysis, it can be concluded the surface sediments are characterized with higher amounts of *n*-alkanes (Fig. 2), and higher CPI and WaxCn(%) than the bottom ones (Fig. 4), which suggests a relatively higher input ratio for the terrestrial plant derived organic matter than the bottom sediments^14^. The sediments form the depth of 30 m to 20 m (S–02 and S–03) have substantially lower *n*-alkanes with lower CPI (close to 1) and WaxCn(%), which suggests a substantial contribution of petroleum derived OM^31^. Therefore, it seems the OM in the study bore cores (S–02 and S–03) can be divided into two types. The first one is likely that immature terrestrial derived OM is present in the surface section of the two boreholes, which is characterized by a muddy layer, with a shift to the deeper sediments. The second one is the thermally mature derived OM, which is dominant in the sediment core deeper than 16 m. The analysis of the lithology indicates that a clay layer locates in the depth of 14–20 m. The CPI and WaxCn (%) do not have depth dependent trend from the depth of 0–16 m. This may indicate the young allochthonous OM may have been delivered to the deeper coarse sands in the form of dissolved organic carbon (DOC) or reworking and secondary sedimentation of ancient, eroded OM during more recent times. However, this process would not change the CPI of *n*-alkanes, which means no substantially biodegradation process during the transport process^14^. Massive groundwater irrigation may lead to surface-derived OM being drawn down into the aquifer systems^36^. In the study area, people rely on groundwater for living and drinking. However, the sampling sties have been used as the farmland for growth of cotton, wheat, and rice. Groundwater may have hydraulic connection with surface water bodies such as ponds, irrigation channel, and wetlands. Precipitation/evaporation together with irrigation using surface water induced seasonal variation of groundwater level, which was high in July-October and low in March–May^11^. Therefore, the transport of fresh terrestrial OM to deeper aquifer system is possible. This process may have been hindered at 16 m depth due to the impermeable character of the clay layer locating in the depth of 14–20 m. This clay layer acts as the transition between terrestrial and petroleum derived OM.

### Bacterial characterization

**Figure 5:**
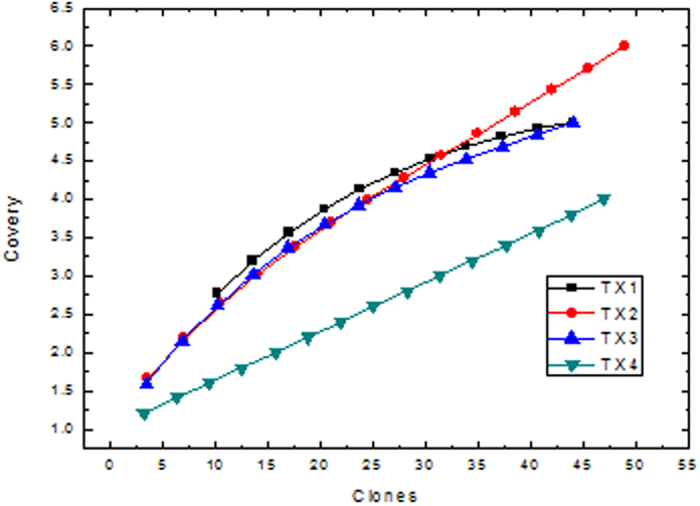
Frequencies of OTUs affiliated with major phylogenetic groups in the bacterial clone libraries of four representative sediment samples.

Four samples (TX1, TX2, TX3 and TX4) with varied As Concentration of 1.5, 6.8, 8.8, 15.0 μg/g, at the depth of 14, 20, and 22 m from S–03, and 10 m from S–02 were selected for community analysis based on 16S rRNA gene clone libraries. It can be seen that the As concentrations in these four samples range from 1.5 to 15.0 μg/g, which represents the low, medium and high As contamination samples. A total 200 bacterial 16S rRNA gene clone sequences were obtained and then subject to BLAST search in NCBI GenBank and phylogenetic analysis. The coverage values of these bacterial clone libraries are 94–98% (Fig. 5). Analysis of 200 cloned sequences from the sediment microbial communities allows us to identify 184 OTUs at a 98% cutoff in samples of TX1–TX4 (Table 1).

Five bacterial groups are present in the higher As sediment sample (TX3, Table 1). In detail, 82% of the identified clones are associated with the *Betaproteobacteria* (4.6%), *Epsilonproteobacteria* (16%) and *Gammaproteobacteria* (61%). This phenomenon is similar to one of the previous studies^37^. Members of *Actinomycetes* (6.8%), *Acidobacteria* (2.3%), *Firmicutes* (6.8%), and *Chloroflexi* (2.2%) were also identified. 94% of the clones obtained are affiliated with the *Alphaproteobacteria* (2.1%), *Epsilonproteobacteria* (11%), and *Gammaproteobacteria* (81%) in sediment with highest arsenic contamination (TX4). Members of *Acidobacteria* (2.1%), *Firmicutes* (2.1%), and *Chloroflexi* (2.1%) were identified too. In the class *Gammaproteobacteria*, 62% of the identified clones are 100% similar to *Thiobacillus thioparus*; 7.6% of the clones are commonly sequenced as genus *Hydrogenophaga*, which was reported as one type of arsenite-oxidizing bacteria isolated from groundwater containing high concentration of As^6^,^38^. 6.8% of the clones belong to genus *Sulfuricella*, which is one type of *chemolithoautotrophic* bacteria growing by the oxidation of sulfur containing components^39^. In the class *Alphaproteobacteria*, 60% of the clones are 100% similar to *Brevundimonas,* which was identified from a freshwater swamp adjoining to Lake Washington^40^. 80% of the clones in class *Epsilonproteobacteria* are 100% similar to *Sulfurimonas autotrophica*, which are chemolithoautotrophic bacteria, by using carbon dioxide as carbon source, and inorganic components containing sulfur for the energy to power their metabolic processes^41^.

### Bacterial community characterization

**Figure 6:**
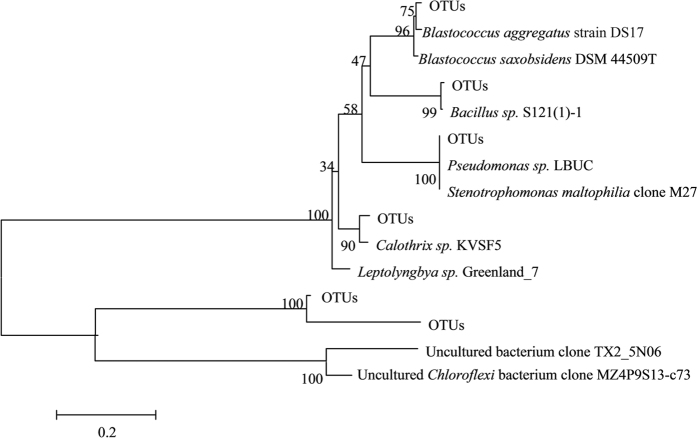
Neighbor-joining trees.

Figure 6 depicts the neighbor-joining trees to classify the bacterial community characterization in the present study. The obtained bacterial 16S rRNA gene clone sequences could be grouped into twelve bacterial phyla: *Alpha-, Beta- Delta-, Epsilon-*, and *Gammaproteobacteria, Chlorobi, Bacteroidetes, Firmicutes, Chloroflexi, Actinobacteria*, and *Deinococus-Thermus*. The bacterial 16S rRNA gene clone libraries are mainly composed of proteobacterial sequences, which accounts for 59%, 61%, and 81% in sample TX2, TX3, and TX4, respectively. The proteobacterial clone sequences are mainly affiliated with *Alpha-, Beta-* and *Gammaproteobacteria* (Table 1). At the phylum level, the relative abundances of major groups vary among samples. At the genus level, *Acinetobacter* and *Pseudomonas* are abundant in all these samples.

The major groups in the TX2 sample include *Acinetobacter, Pseudomonas, Brevundimonas, Aquabacterium, Psychrobacter, and Geobacter* with abundant *Pseudomonas* and *Acinetobacter* (10% and 59%, respectively). The TX3 sample consists of seven major genera, including *Acinetobacter, Pseudomonas, Brevundimonas, Massilia, Dietzia, Sphiingomonas*, and *Planococcus, where* the former four are dominant (5%, 7%, 7% and 16%, respectively). The TX1 sample is composed of four major genera, including *Acinetobacter, Pseudomonas, Aquabacterium*, and *Arthrobacter, where* the former two are dominant (7% and 23%, respectively). Previous studies have shown that some of the above identified genera could involve in As cycling. For example, some *Acinetobacter* strains are more resistant to arsenic than other species, and some of them can even oxidize or reduce arsenic^42^. In addition, some of *Brevundimonas spp., Massilia spp., Dietzia spp. and Planococcus spp.* are capable of reducing arsenate and/or resisting As. And *Dietzia* was specially reported as the type species in the extreme environment of hyper-alkaline and hyper-saline soil and water contaminated by heavy metals^43^.

16S rRNA clone library search indicate that *Acidobacteria, actinomycetes* and *Hydrogenophaga*, who are similar to those As resistant bacteria^42^, are dominated in the sediments containing high concentration of As in the present study. The release of dissolved As, and Fe from sediment to groundwater might be due to the reductive dissolution of Fe-oxyhydroxides by the microbial populations found within the sediment in the reducing environment, because the reductive metals (e.g., As (III) and Fe (II)) are more soluble in water and less affinitive to sediment than their oxidative species.

## Conclusions

Arsenic was abundant in sand and top soil, but low in clay. *n*-alkanes were abundant in the surface sediments (0–10 m), but low in deeper section in the three boreholes. The analysis of corresponding diagnostic ratios (e.g., CPI and WaxCn (%)) of *n*-alkanes suggested that OM in shallow sediment was mainly derived from fresh terrestrial plants; but they were mainly from petroleum hydrocarbons in deeper section of the bore holes. OM derived from different sources may facilitate the release of As from aquifer system to groundwater. The shift of the concentration of As, *n*-alkanes and CPI values along the sediment depth reflected the sediment lithology. The 16S rRNA gene sequences extracted from the sediments suggested that diverse bacterial communities were present in the aquifers with the contamination of arsenic; microbial community structures with different arsenic concentrations were quite different. The microbial communities were abundant with *Acidobacteria, Actinomycetes* and *Hydrogenophaga* in the studied sediments with high concentration of arsenic. These results implied that microbes and OM derived from different sources may play an important role in arsenic mobilization in the aquifers of Jianghan Plain, Hubei, China.

## Additional Information

**How to cite this article:** Ye, H. *et al*. Sediment biomarker, bacterial community characterization of high arsenic aquifers in Jianghan Plain, China. *Sci. Rep.* **7**, 42037; doi: 10.1038/srep42037 (2017).

**Publisher's note:** Springer Nature remains neutral with regard to jurisdictional claims in published maps and institutional affiliations.

## Figures and Tables

**Table 1:** Bacterial community structure based upon the 16S rRNA genes.

	TX1	TX2	TX3	TX4
*Actinomycetes*	11%	2.0%	6.8%	/
*Acidobacteria*	4.6%	2.0%	2.3%	2.1%
*Cyanobacteria*	2.3%	2.0%	/	/
*Chloroflexi*	/	2.0%	2.3%	2.1%
*Firmicutes*	4.6%	10%	6.8%	2.1%
*A-proteobacteria*	6.8%	/	/	2.1%
*B-proteobacteria*	23%	18%	4.6%	/
*E-proteobacteria*	/	4.1%	16%	11%
*G-proteobacteria*	48%	59%	61%	81%

Notes: “/” indicates non-available. TXi (i = 1, 2, 3, 4) represents four sediment samples with different arsenic levels from the boreholes of S–02 and S–03.
